# Anterolateral advancement pharyngoplasty versus barbed reposition pharyngoplasty in patients with obstructive sleep apnea

**DOI:** 10.1007/s00405-023-08402-1

**Published:** 2024-01-03

**Authors:** Tarek Abdelzaher Emara, Mohamed Sherif Ahmed Abd Elmonem, Ashraf Mahmoud Khaled, Hisham Ahmed Hasan Genedy, Rabie Sayed Youssef

**Affiliations:** 1 Faculty of Medicine, Zagazig University, Zagazig, Egypt; 2 Faculty of Medicine, Beni-Suef University, Beni Suef, Egypt; 3 Faculty of Medicine, ENT Department, Beni-Suef University, Beni Suef, Egypt

**Keywords:** Palatopharyngeus, Pharyngoplasty, Apnea

## Abstract

**Objectives:**

To compare functional outcomes and complication rates of anterolateral advancement pharyngoplasty (ALA) versus barbed reposition pharyngoplasty (BRP) in the treatment of obstructive sleep apnea patients with palatal and lateral pharyngeal wall collapse.

**Study design:**

Prospective study.

**Setting:**

University hospitals.

**Subjects and methods:**

Forty-six patients were included in this study. Patients were divided into two groups randomly, group 1 (23 cases) underwent anterolateral advancement pharyngoplasty and group 2 (23 cases) underwent barbed relocation pharyngoplasty. According to the following criteria: both sex, age between 18 and 65 years, body mass index ≤ 32 kg/m2, Friedman stage II or III, type I Fujita, nocturnal polysomnography study diagnostic for OSA, retropalatal and lateral pharyngeal wall collapse, diagnosis with flexible nasoendoscopy during a Muller’s maneuver based on a 5-point scale and drug-induced sleep endoscopy. Patients who suffered from retroglossal airway collapse were rolled out.

**Results:**

Apnea–hypopnea index decreased from 27.50 ± 11.56 to 11.22 ± 7.63 (*P* ≤ .001) in group 1 and from 33.18 ± 10.94 to 12.38 ± 6.77 (*P* ≤ .001) in group 2. Retropalatal posterior airway space increased from 9.84 ± 1.29 mm to 21.48 ± 2.8 mm (*P* ≤ .001) in group 1 and increased from 10.26 ± 1.2 mm to 22.86 ± 2.62 mm (*P* ≤ .001) in group 2. Retropalatal space volume increased from 1.9 ± 0.68 cm^3^ to 2.75 ± 0.7 cm^3^ (*P* ≤ .001) in group 1 and increased from 1.96 ± 0.88 cm^3^ to 2.82 ± 0.83 cm^3^ (*P* ≤ .001) in group 2. Surgical success was 86.95% in group 1 compared to 82.6% in group 2.

**Conclusions:**

Both techniques appear to be effective with a high surgical success rate in the treatment of OSA patients with retropalatal and lateral pharyngeal wall collapse.

**Supplementary Information:**

The online version contains supplementary material available at 10.1007/s00405-023-08402-1.

## Introduction

Being a public health problem, obstructive sleep apnea–hypopnea syndrome predominance ranges between 9% and 38% with an Apnea–Hypopnea Index (AHI) greater than 5 [1]. However, it is highly underdiagnosed as the OSA is not always accompanied by daytime sleepiness which can leave the sleep-disordered breathing unnoticed [2].

The etiology of OSA is multifactorial, consisting of a complex interplay between anatomic, neuromuscular factors and an underlying genetic predisposition toward the disease [3].

Obstructive sleep apnea is presented as frequent complete or partial closure of the upper airway during sleep resulting in sleep fragmentation and oxygen desaturation [4].

The collapse of the upper airway is usually multilevel. Collectively, oropharynx, velum, and lateral pharyngeal walls obstruction are the most common sites of obstruction in multi-level obstruction patients. The oropharynx and lateral pharyngeal walls are the most common site of obstruction as a single site of obstruction [5, 10].

The lateral pharyngeal wall (LPW) is a relatively complex structure composed of different groups of pharyngeal muscle and lymphoid tissue (palatine tonsils). The palatopharyngeus muscle (PPM) is one of the most critical muscles of the LPW and soft palate. Gray’s Anatomy stated that within the soft palate, the PPM consists of anterior and posterior fasciculi, which are attached to the superior surface of the palatine aponeurosis. Those two fasciculi are separated from each other by the levator veli palatine muscle (LVP) although lying in the same plane. In patients with OSA, the thickness and collapsibility of LPW is more than that of normal individuals when subjected to airflow pressure, LPW narrowing appears to be one of the most important oropharyngeal findings that carries a risk factor for male OSA patients [6, 7, 10].

The most efficient and widely used treatment for OSA is continuous positive airway pressure (CPAP), which has been available since the 1980s [11].

In selected patients, who are not compliant with CPAP therapy, and whose main site of obstruction is palatal or lateral pharyngeal wall collapse, surgical management with pharyngoplasty techniques can be offered. The main object of this surgical technique is to improve upper airway measurements by reducing soft-tissue collapsibility to be curative, or at least to improve compliance to CPAP therapy [12, 13].

Uvulopalatopharyngoplasty (UPPP) was first described by Ikematsu in 1964, then Fujita introduced it in the USA in 1981 [14, 15]. The role of this procedure has been questioned, in the last 2 decades many surgeons started developing newer techniques to address the palate for OSA patients, which seems to have a better result in the long term [16, 17].

Barbed suture is a type of knotless suture that has many barbs on its surface, while conventional sutures depend mainly on a surgeon's ability to tie secure knots, these barbs penetrate deep inside the tissue and fix them into place, eliminating the need for knots to tie the suture [18, 19]. In 2013, Mantovani et al. considered the possibility of utilizing self-locking threads to perform Barbed Roman Blind pharyngoplasty [20], as a modification of his original Roman Blind Pharyngoplasty, 2012 [21]. Then, Vicini et al. developed a new variant of ESP using the barbed sutures, Barbed reposition pharyngoplasty (BRP) in 2015 [22]. In 2016, Emara et al. described anterolateral advancement pharyngoplasty (ALA) with no tissue resection nor muscle cutting at all, except for tonsillectomy if not performed before [10].

The recent advances considering surgical techniques of pharyngoplasty aimed to gain the expansion and stabilization of the pharyngeal airway through the conservative management of LPW collapse rather than through the excision of excess pharyngeal soft tissue [16, 23]. Both BRP and ALA techniques target the soft palate and lateral pharyngeal wall of the oropharynx. These techniques aimed to much less tissue resection rather than through ablation of excess pharyngeal soft tissue [10, 22]. 

The objective of this study is to compare the functional outcomes, efficacy, safety, feasibility, and complications of anterolateral advancement pharyngoplasty versus barbed reposition pharyngoplasty in the treatment of OSA patients with palatal and lateral pharyngeal wall collapse.

### Study design

From July 2019 to September 2021, 46 OSA patients (age range, 21–60 years) from the Department of Otolaryngology-Head and Neck Surgery, Zagazig University Hospitals, Beni Suef University Hospitals, and private practice, Egypt, were included in this study. Patients were randomly divided into two groups, patients allocation were set using computer software (Microsoft Excel), to generate random numbers of subjects and their assortment, group 1 (23 cases) underwent anterolateral advancement pharyngoplasty (ALA group) and group 2 (23 cases) underwent barbed relocation pharyngoplasty (BRP group).

### Sample size

The sample size was calculated using the following formula [24]:

$$n = 2\left[ {\frac{{\left( {Z_{\alpha /2} + Z_{\beta } } \right) \times \sigma }}{{\mu_{1} - \mu_{2} }}} \right]$$

where:

*n* = sample size

*Z*_α/2_ = 1.96 (The critical value that divides the central 95% of the Z distribution from the 5% in the tail)

*Z*_*β*_ = 0.84 (The critical value that separates the lower 20% of the Z distribution from the upper 80%)

*σ* = the estimate of the standard deviation of AHI (Apnea–Hypopnea Index) = 1.8

*µ*_1_ = mean AHI post-Anterolateral–advancement–pharyngoplasty = 16.3. [10]

*µ*_2_ = mean AHI post-Barbed–reposition–pharyngoplasty = 13.57 [22]

Therefore, by calculation, the sample size was equal to 11 subjects per group at least, giving a total sample size of 22 subjects after addition of 10% drop-out proportion; however, 46 subjects were included in the final work.

### Inclusion criteria

Clinical picture suggestive of OSA, the degree of OSA, classified according to the clinical guidelines given by the American Academy of Sleep Medicine [25], having the main site of obstruction at the retropalatal level and lateral pharyngeal wall.; both sex; small tonsils (tonsil-size 1–2); BMI ≤ 32 kg/m^2^; Friedman clinical stage II and III [26]; nocturnal polysomnography study diagnostic for OSA, retropalatal and lateral pharyngeal collapse, diagnosis with flexible nasoendoscopy during a Muller’s maneuver based on a 5-point scale and drug-induced sleep endoscopy (DISE); and incompetence to tolerate continuous positive airway pressure therapy or for whom such treatment failed. Patients with central sleep apnea, retroglossal airway collapse, and previous history of tonsillar and palate surgery were excluded from the study.

The study was conducted after approval from the research ethical committee, Faculty of Medicine, Beni Suef University, approval No: FMBSUREC/03092019 and written informed consent was obtained prior to surgery from all patients.

### Preoperative assessment

Clinical examination, history taking, Epworth Sleepiness Scale (ESS) evaluation, cephalometry, CT volumetric assessment for the upper airway to evaluate the retropalatal space, and nocturnal polysomnography. Flexible nasoendoscopy was done for all patients, and collapse during Muller’s maneuver was scored for the LPWs, soft palate, and base of the tongue on a 5-point scale. In addition, drug-induced sleep endoscopy (DISE) was evaluated using the VOTE scoring system [27]. 

### Study population

Forty-six patients were divided into two groups randomly, patients were assigned using computer software (Microsoft Excel), to generate random numbers of subjects and their assortment, group 1 (23 cases) underwent anterolateral advancement pharyngoplasty and group 2 (23 cases) underwent barbed relocation pharyngoplasty.

### Hardware

Surgical instruments used for both procedures were the same except for the suture material, i.e., Dingman mouth gag, bipolar cautery forceps, needle holder, and scissors. The specific device for ALA procedure was the Vicryl thread while for BRP was the barbed thread. Among the available types of barbed threads, we chose the BARBED BIOLIFT thread by Taisier-Med Inc, EGYPT.

### Surgical techniques

Each surgical procedure was performed under general anesthesia by the same surgeon with either nasotracheal or orotracheal intubation. The patient was placed in a supine position with an extended head, and a Dingman mouth gag was placed to adequately expose the oropharynx. Starting with measuring retropalatal space in two dimensions and the length of the uvula with a ruler.

**Fig. 1:**
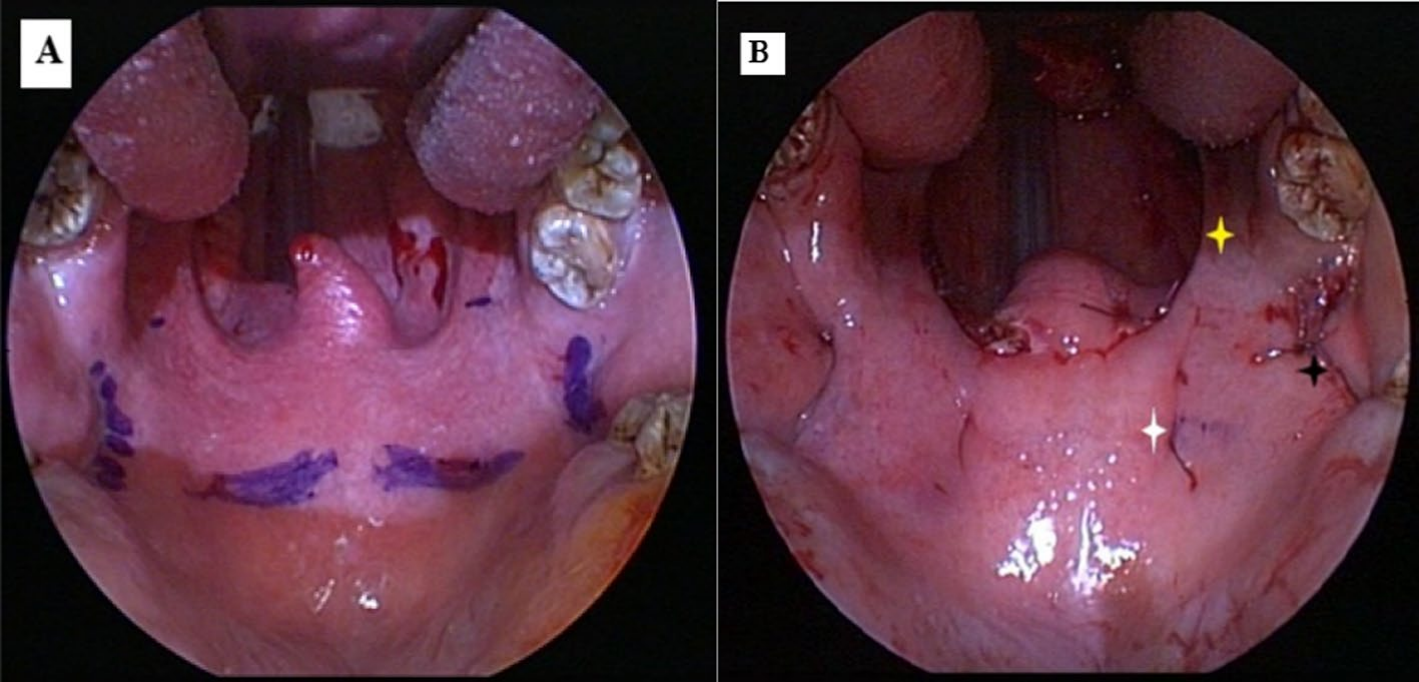
(Group 1) Anterolateral Advancement Pharyngoplasty **A** Preoperative view **B** postoperative view  First suture between PPM and PMR.  Second suture between PPM and LVP.  Third suture between PPM and SPC

*Group (1) Anterolateral Advancement Pharyngoplasty (ALA) According to Emara et al., Technique (2016). *Fig. 1.

Starting with bilateral tonsillectomy, sparing the palatopharyngeus muscle (PPM) and the mucosa of both tonsillar pillars with meticulous dissection of both fasciculi of the PPM and the superior pharyngeal constrictor (SPC) muscles (without violating their muscular fascicules).

It is critical to not violate the connecting fibrous tissues between the anterior part of PPM and SPC muscles and preserve this connection.

Within the tonsillar fossa, the SPC muscle was grasped and plicated with 2–0 Vicryl through 2 mattress-style sutures. The main PPM directly just below the confluence of both fasciculi and the plicated SPC were sutured with a 0 Vicryl thread, which is then raised and advanced to be fixed to the pterygomandibular raphe (PMR) through a ‘‘figure-of-eight suture’’ to achieve anterolateral expansion.

Attention was then given to the posterior part of the PPM; 3–0 Vicryl suture was passed and tightened around its muscular fasciculus near the midline of the soft palate and base of the uvula. The posterior part of the PPM, together with its attached mucosa, was then advanced superolaterally behind the palatoglossus muscle (PGM) to be hooked up to the levator veli palatini (LVP) muscle of the same side 15 mm above the free edge of the palatine arch by a f ‘figure-of-eight suture’’ suture.

In doing so, the over-elongated uvula shortened without removal of any of its structure. The inferior half of the PPM was laterally sutured to the SPC through 2 mattress-style sutures. Achieving such advancement of both the PPM and the SPC will obtain a steady anterolateral fixation, which moves the soft palate and LPW in a forward direction and creates an instant increase of oropharyngeal anteroposterior and lateral dimensions. We did not incise or remove any mucosa, suture tonsillar pillars, or any part of the uvula. On the other side, the same steps were performed. Operative time was calculated using a stopwatch.

**Fig. 2:**
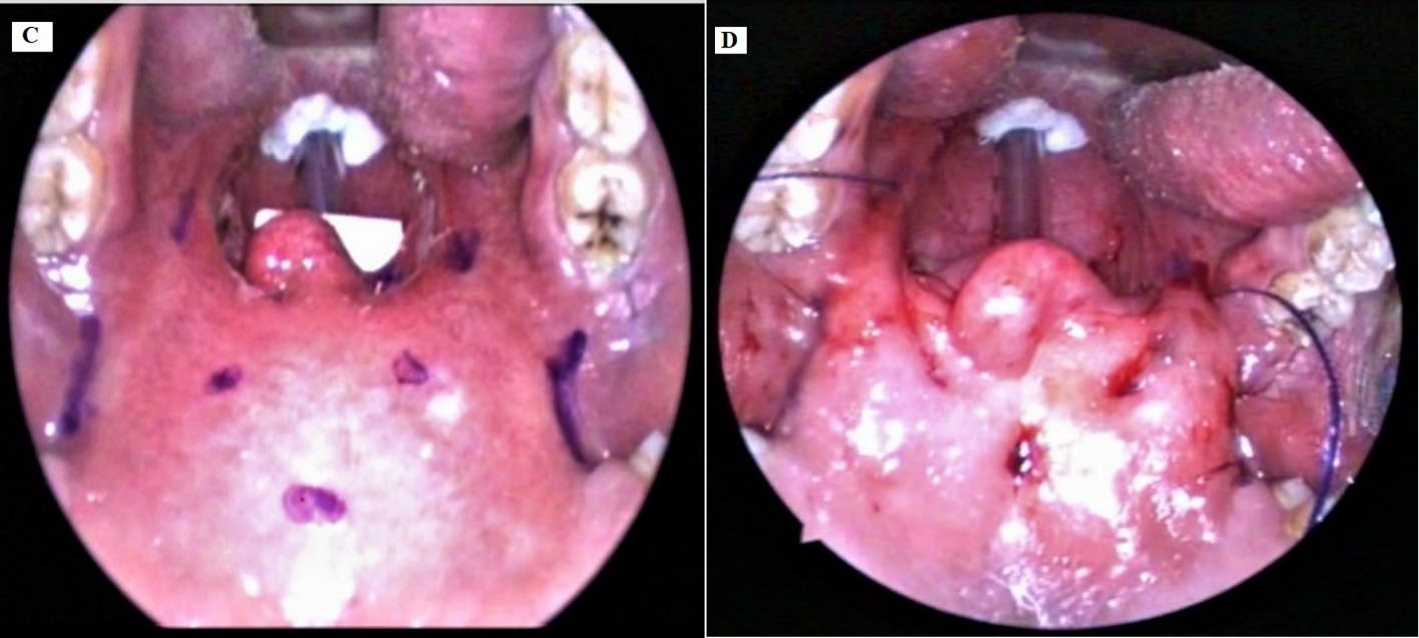
(Group 2) Barbed relocation pharyngoplasty **C** preoperative view, **D** postoperative view

*Group (2) Barbed Relocation Pharyngoplasty (BRP) According to Vicini et al., Technique (2015).* Fig. 2*.*

Starting with bilateral tonsillectomy, identification, and meticulous sparing of the PPM and PGM, with sparing as much as possible of the mucosal covering of both pillars. Two weakening partial incisions were done at the inferior part of the PPM.

A full-thickness triangle (muscle and mucosa) was excised at the superolateral angle of the tonsil to achieve a wider and furthermost squared oropharyngeal inlet. The center of the palate at the palatal spine and PMR were marked.

Using a single barbed suture, bidirectional polydioxanone absorbable monofilament, size 2/0, with a transition zone in the middle. One needle was introduced at the center point and then passed laterally within the palate, turning around PMR till it comes out at the most superior part of the raphe at one side. The needle again was re-introduced close to point of exit, passing around the PMR, till it comes out into the tonsillectomy bed, then through the upper part of the PPM and emerges close to the mucosa of the posterior pillar, does not penetrate through it. The posterior pillar was entered at the junction between the upper third and the lower two-thirds. Then, again the needle was passed back through the tonsillectomy bed and then this suture will be suspended around the raphe again; gentle traction on the thread, and no knots were taken. This results in a stable relocation of the posterior pillar to a more lateral and anterior position, after which this suture was repeated at least three times till the lower pole of the muscle had reached. The opposite side was done in the same way. The tip of the uvula was not removed if it is short. Operative time was calculated using a stopwatch.

The principle of those two techniques is to advance and fix the PPM to the PMR. The anesthesiologist was performing late extubation. The patient stayed under close observation during the early postoperative period, in ICU or intermediate care for monitoring any postoperative complications.

### Statistical analysis

SPSS version 16 was used to statistically analyze collected data (SPSS, Inc, an IBM Company, Chicago, Illinois). Mean and standard deviation were used to summarize data, and the paired *t* test was used for testing the difference between paired data. Probability was considered significant if *P* was ≤ 0.005.

## Results

The age, sex, and BMI of all patients were shown in Table 1. There was no statistically significant difference between the studied groups as regards the demographic data and BMI (*P* value > 0.05).

**Table 1:** Age, sex and BMI

	ALA group *N* = 23	BRP group *N* = 23	*P* Value
Age (range) Mean ± SD	21–59 (43.21 ± 11.61)	25–59 (47 ± 8.4)	> 0.05
Sex			> 0.05
Male	14 (60.86%)	17 (73.91%)	
Female	9 (39.13%)	6 (26.08%)	
BMI (range) Mean ± SD	24.9–32 (30.14 ± 1.8)	26.6–32 (30.8 ± 1.62)	> 0.05

**Fig. 3:**
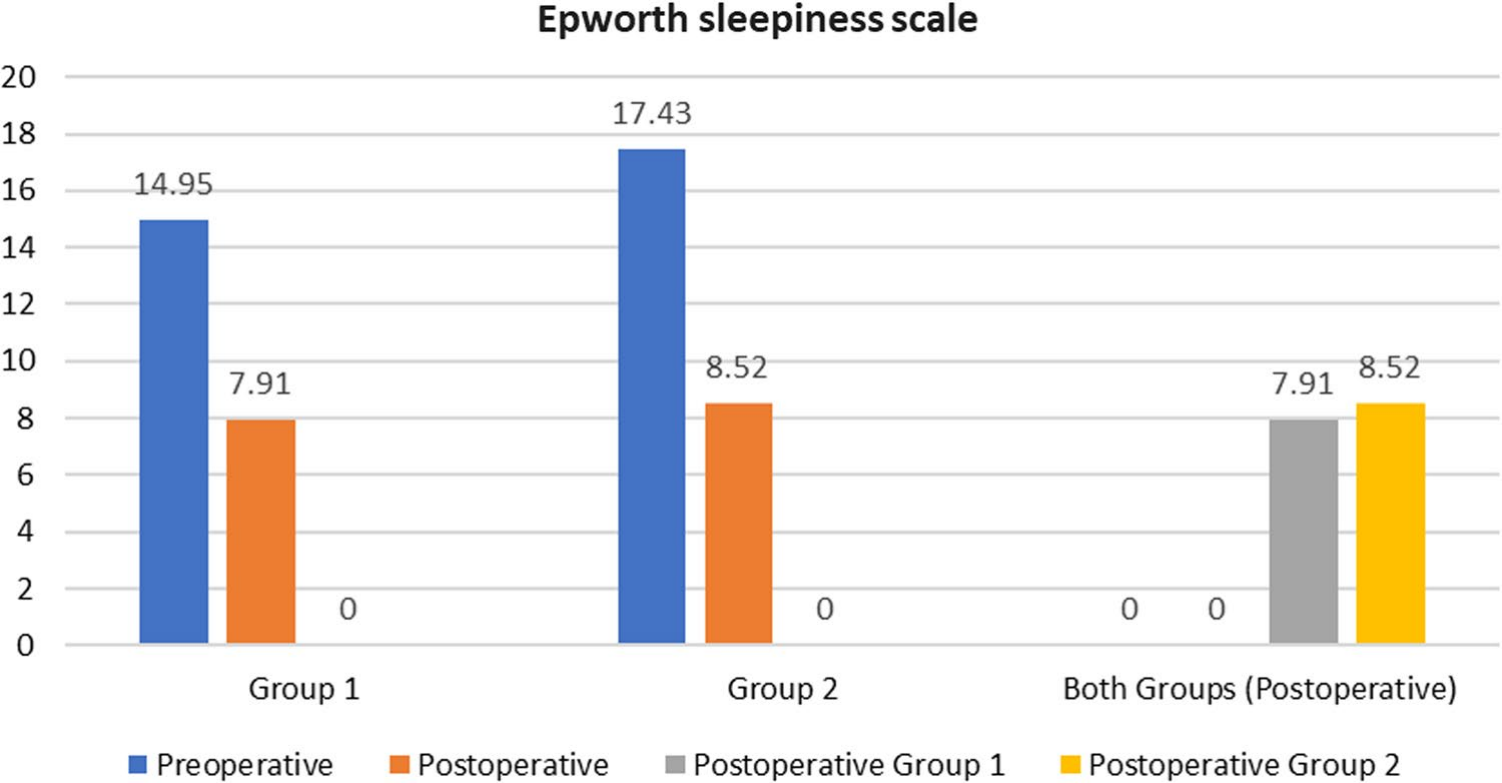
Column chart showing preoperative and postoperative ESS among patients in both groups

**Fig. 4:**
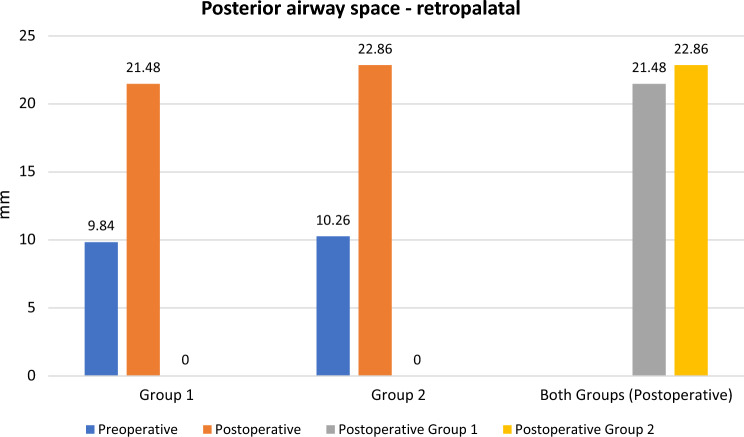
Column chart showing preoperative and postoperative PAS-t among patients in both groups

**Fig. 5:**
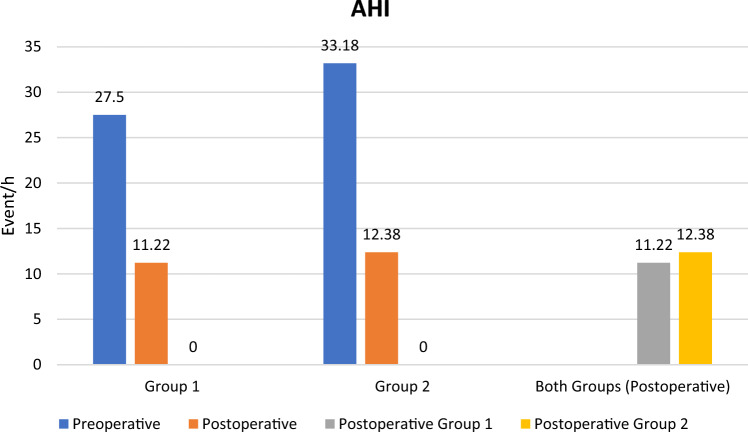
Column chart showing preoperative and postoperative AHI among patients in both groups

The preoperative and postoperative Epworth sleepiness scale (ESS), apnea–hypopnea index (AHI), lowest O2 saturation (LOS), posterior airway space (PAS) at the level of the soft palate (retropalatal-PAS; PAS-t), soft palate length, soft palate thickness, retropalatal space volume, snoring index, sleep efficacy and VAS for snoring were shown in Table 2. There was a highly statistically significant difference between preoperative and postoperative results (*p* value < 0.001) in each group, while there was no statistically significant difference between postoperative results in both groups (*p* value > 0.05) Figs. 3, 4 and 5.

**Table 2:** Preoperative and postoperative major study parameters

	ALA group (*n* = 23)			BRP group (*n* = 23)			*P* value
	Preoperative	Postoperative	*P* Value	Preoperative	Postoperative	*P* Value	ALA Vs. BRP
ESS (range) Mean ± SD	7–21 14.95 ± 3.72	3–16 7.91 ± 3.05	< 0.001	8–24 17.43 ± 4.47	3–18 8.52 ± 3.67	< 0.001	> 0.05
AHI (range) Mean ± SD	15.8–57.8 27.50 ± 11.56	4–21 11.22 ± 7.63	< 0.001	16.1 to 53 33.18 ± 10.94	3–22 12.38 ± 6.77	< 0.001	> 0.05
LOS, % (range) Mean ± SD	65–89 81.86 ± 6.41	82–95 90.21 ± 3.70	< 0.001	68–87% 81.75 ± 6.42	82–91% 88.60 ± 2.31	< 0.001	> 0.05
PNS-P, mm (range) Mean ± SD	33.4–45.6 40.84 ± 4.54	26.7–38.1 30.52 ± 2.6	< 0.001	34.9–44.7 38.67 ± 3.11	25.1–34.4 29.49 ± 2.14	< 0.001	> 0.05
Point G, mm (range) Mean ± SD	10.3–15 13.04 ± 1.42	7.1–11.4 8.88 ± 1.08	< 0.001	9.56–16.7 12.68 ± 2.07	7.1—10.9 8.93 ± 1.33	< 0.001	> 0.05
PAS-t, mm (range) Mean ± SD	8–13.2 9.84 ± 1.29	17.6–27.6 21.48 ± 2.8	< 0.001	8.1–12.69 10.26 ± 1. 2	16.89–26 22.86 ± 2.62	< 0.001	> 0.05
Sleep Efficacy, % (range) Mean ± SD	59.6–92 81.21 ± 10.89	65.2–96 88.28 ± 8.72	< 0.001	50.7–89 86.32 ± 12.58	70.2–93 92.87 ± 6.48	< 0.001	> 0.05
RP CT volume, cm^3^ (range) Mean ± SD	0.9–3.21 1.9 ± 0.68	1.7–3.79 2.75 ± 0.7	< 0.001	0.66–3.6 1.96 ± 0.88	1.02–3.84 2.82 ± 0.83	< 0.001	> 0.05
RP axial AP, mm (range) Mean ± SD	8.6–14.0 10.68 ± 1.37	17.5–28.2 22.36 ± 2.78	< 0.001	8.18–13.82 10.42 ± 1.41	17.1–27.6 23.54 ± 2.73	< 0.001	> 0.05
Snoring Index, /h (range) Mean ± SD	111–412 244.24 ± 82.38	0–231 99.95 ± 71.52	< 0.001	148.3–706.5 331.79 ± 151.57	0–487 111.06 ± 66.4	< 0.001	> 0.05
VAS Snoring Mean ± SD	5.3 ± 1.39	1.6 ± 1.15	< 0.001	4.86 ± 1.6	1.47 ± 0.89	< 0.001	> 0.05

*ESS* Epworth sleepiness scale, *AHI* apnea–hypopnea index, *LOS* lowest oxygen-saturation level, *PNS-P* soft palate length, *Point G* soft palate thickness, *PAS-t* retropalatal posterior airway space, *RP* retropalatal, *AP* anteroposterior, *VAS* visual–analogue scale.

Operative time was ranged from 17 to 31 min (23.78 ± 4.84) in group 1 and ranged from 17 to 24.8 min (22.01 ± 3.06) in group 2, with no statistically significant difference between both groups (*P* value > 0.05). The surgical success rate was defined by postoperative AHI decrease by 50% and/or AHI below 20 [28] according to this definition; there were (20/23) successful patients (86.95%) in group 1 with three failed patients, compared to (19/23) successful patients (82.6%) in group 2 with four failed patients, with no significant statistical difference between both groups (*P* value > 0.05).

The main postoperative complications were foreign body sensation, dysphagia, and pain. Foreign body sensation gradually improved within two months while dysphagia and pain gradually improved within 1 month. Foreign body sensation and dysphagia showed statistically significant differences between both groups. The mean of pain measurements according to visual–analog scale (VAS) were shown in Table 3. There was a highly statistically significant difference between preoperative and postoperative VAS for pain in each group (*P* value < 0.001), while there was no statistically significant difference between postoperative VAS for pain in both groups (*P* value > 0.05). As regard extrusion of the suture, there was no statistically significant difference between both groups (*P* > 0.05). All these complications improved gradually or were treated medically. As regard temporarily postoperative velopharyngeal insufficiency which gradually improved within one month, there was no statistically significant difference between both groups (*P* > 0.05), the Mean absorption time for the barbed suture was about 180 days.

**Table 3:** Postoperative complications and pain

	Group 1 (ALA)	Group 2 (BRP)	*P* value
Foreign body sensation (Range) (Mean ± SD)	4–21 (8.78 ± 5.96)	11–60 (25.95 ± 17.40)	< 0.005
Dysphagia (Range) (Mean ± SD)	4–15 (8.17 ± 3.55)	4–27 (15 ± 6.40)	< 0.004
VAS for pain (Mean ± SD)			> 0.05
First week	4.6 ± 1.03	5.13 ± 1.71	
Second week	1.04 ± 1.29	1.56 ± 1.37	
One month	0	0.17 ± 0.51	
Extrusion of suture	0 (0%)	2 (8.69%)	> 0.05
Temporarily VPI	0 (0%)	2 (8.69%)	> 0.05

## Discussion

Both anterolateral advancement (ALA) pharyngoplasty and barbed reposition pharyngoplasty (BRP) aimed to achieve a surgical success for the treatment of OSA patients with both antero-posterior and lateral wall collapse at the velopharynx. Thus, in this study, we compared results of ALA pharyngoplasty and BRP to prove which technique allows the best functional outcomes in OSA patients with isolated retropalatal collapse.

Both ALA pharyngoplasty and BRP aimed to suspend PPM to PMR as an anchor point to suspend the soft palate and LPW. However, the main differences between ALA pharyngoplasty and BRP are:

- Removal of a full thickness triangle at the supratonsillar area in BRP.
- Two releasing partial incisions to the PPM in BRP.
- Partial removal of the elongated uvula may be needed in BRP.
- Splinting of the LPW through plication of PPM and SCP by 2 mattress-style sutures to decrease LPW collapse in ALA pharyngoplasty. Because the origin of the SPC muscle in the tonsillar fossa region is more lateral (mandibular mylohyoid line) than the PPM, subsequently suturing the 2 muscles and fixing them to the PMR intends to provide anterolateral support to the oropharynx and tongue base.
- Involvement of LVP muscle in ALA pharyngoplasty through hanging up the posterior part of PPM along with its attached posterior pillar mucosa with a figure-of-eight suture to it, which ensures shortening of the elongated uvula without the need to excise any of its structure.

The knotless barbed suture is an innovative and relatively new type of suture [29]. Barbed sutures are conceived to distribute tension along the full length of the thread route and to create dynamic vectors inside the soft tissue without the necessity of knots and avoiding subsequent ischemic damage [19, 30].

In a multicenter prospective study performed with 111 patients, Montevecchi et al. reported that the success rate was 73% for Barbed pharyngoplasty (BP) [31]. However, promising results have been reported by Vicini et al. with a success rate of 90% [32] . In 2017, Cammaroto et al. showed similar results in patients with multilevel OSA at retropalatal and retrolingual airway collapse treated with palatal surgery combined with transoral robotic surgery (TORS). The study showed no major difference between the BP and the Expansion sphincteric pharyngoplasty (ESP) groups, although both techniques proved to be more effective than UPPP in a multilevel setting. However, BP was seen to be a quicker and easier technique and provided minimal blood loss and better preservation of the mucosal and muscular tissues in comparison with ESP and, of course UPPP [33]. Babademez et al. 2020 also showed a success rate of 86.6% for BRP in a study on 129 patients with mild-to-moderate OSA [34]. On the other hand, Emara et al. 2016 showed a success rate of 86.8% for ALA pharyngoplasty in a study on 41 patients with mild-to-severe OSA [10]. In our study, we reported a success rate of 86.95% in ALA group and 82.6% in BRP group with no significant statistical difference between both groups.

Our results showed that the mean ESS significantly improved by ~ 50% in both ALA and BRP groups (*P* < 0.001) with a comparable results to a study conducted by Babademez et al. 2020 showing improvement by ~ 68% in both BRP and ESPwAP (expansion sphincter pharyngoplasty with anterior palatoplasty) groups [34]. In addition, a study by Montevecchi et al. 2018 showed improvement by about 40% regarding BRP procedure [31], while a study by Vicini et al. 2017 showed improvement by ~ 67% regarding BRP procedure [32].

The mean AHI in our study significantly dropped by ~ 59% and ~ 63% in ALA and BRP groups, respectively, with a comparable results to Babademez et al. 2020 study (~ 68% and ~ 71% in ESPwAP and BRP groups, respectively) [34], Vicini et al. 2017 study (~ 48% in BRP group) [32], Montevecchi et al. 2018 study (~ 60% in BRP group) [31], and Kamel et al. 2023 study (~ 53% and ~ 65% in the UPPP and BRP groups, respectively) [35].

At the same time, mean PAS-t significantly increased by ~ 118% and ~ 123% in ALA and BRP groups, respectively, due to decrease in both soft palate length which significantly decreased by ~ 25% and ~ 24% in ALA and BRP groups, respectively, and soft palate thickness which significantly decreased by ~ 31% and ~ 32% in ALA and BRP groups, respectively. This finally led to increase retropalatal space volume by ~ 44% in both groups and consequently decrease the airway resistance and improvement in the glossopalatal contact (a significant factor in worsening of obstructive events). The improvement of such a contact will leads to an increase in surgical success. These results were not found in any other studies for comparison in the literature.

ALA pharyngoplasty was associated with less pain as measured by VAS score, less dysphagia, less foreign body sensation at the throat postoperatively, and consequently a rapid return to normal diet than BRP. This can be attributed to less tissue resection and less palatal edema. Also, ALA pharyngoplasty was associated with fewer postoperative complications such as palatal edema and palatal bleeding. On the other hand, BRP associated with increased incidence of temporarily postoperative VPI development than ALA pharyngoplasty which improved gradually within one month. This can be attributed to a significant decrease in soft palate length, greater postoperative pain, and dysphagia. BRP was also associated with more liability for suture extrusion than ALA pharyngoplasty.

Regarding operative time, it was less in BRP group than ALA group (22.01 ± 3.06 and 23.78 ± 4.84 min, respectively,* p* > 0.05), these results were comparable to result of Babademez et al. 2020 study where it toke 22.1 ± 13.5 min in BRP group [34], and 25 ± 4.2 min in BRP group in Montevecchi et al. 2018 study [31]. While regarding cost, barbed suture used in BRP is more expensive than Vicryl suture used in ALA pharyngoplasty by about 28.57 folds with a statistically significant difference.

There is no statistically significant difference between both techniques regarding hospital stay, feasibility, and teachability. In our study, 2 patients (8.69%) in BRP group suffered from partial thread extrusion while in Montevecchi et al. 2018 study, 3 cases (3%) suffered from partial thread extrusion during intraoperative period and 7 cases (6%) during postoperative period [31]. 

ALA can be used safely in revision pharyngoplasty and in patients with a previous tonsillectomy while BRP could be used with caution in such cases with the likelihood of a higher chance of VPI and barbed extrusion because there is no enough thickness in the soft palate to contain these threads, and there is also a possibility that when we pass these threads in such a thin palate may deviate from its correct path within the palate tissue during surgery. Regarding this debatable point, we recommend conducting a study from multiple centers in this regard.

The main limitations of our study include a relatively small sample size, CT volume measuring was done by manual tracing which may lead to some statistical bias, but we tried to avoid this by fixing the radiologists who take the measurements and take these measurements twice, then the means were used in the statistical analysis. Also, this study represents a single research center and surgeries performed by the same surgeon, and multicenter studies with different surgeons are required for more statistical results. Finally, this study only demonstrated the short-term results, after 6 months. Ideally, ESS and AHI should be evaluated during the next 5 to 10 years after surgery to conclude whether the favorable effects remain over time.

In conclusion, as offered throughout this study, both techniques, ALA pharyngoplasty and BRP, have relatively high success rates, improvement in ESS, and PSG parameters and also increased posterior airway space. Both techniques were safe with few minor complications. Neither of our cases had major complications such as bleeding and airway compromise.

### Supplementary Information

Below is the link to the electronic supplementary material.

Supplementary file1 (MP4 12,427 KB)

## Data Availability

All data generated during this study are contained in this published article.
